# Quantitative Assessment of Whole-Body Tumor Burden in Adult Patients with Neurofibromatosis

**DOI:** 10.1371/journal.pone.0035711

**Published:** 2012-04-27

**Authors:** Scott R. Plotkin, Miriam A. Bredella, Wenli Cai, Ara Kassarjian, Gordon J. Harris, Sonia Esparza, Vanessa L. Merker, Lance L. Munn, Alona Muzikansky, Manor Askenazi, Rosa Nguyen, Ralph Wenzel, Victor F. Mautner

**Affiliations:** 1 Department of Neurology and Cancer Center, Massachusetts General Hospital, Boston, Massachusetts, United States of America; 2 Department of Radiology, Massachusetts General Hospital, Boston, Massachusetts, United States of America; 3 Department of Radiology, Corades, S.L., Majadahonda, Spain; 4 Department of Radiation Oncology, Massachusetts General Hospital, Boston, Massachusetts, United States of America; 5 Biostatistics Center, Massachusetts General Hospital, Boston, Massachusetts, United States of America; 6 Department of Cancer Biology, Dana-Farber Cancer Institute, Boston, Massachusetts, United States of America; 7 Department of Biological Chemistry, The Hebrew University of Jerusalem, Jerusalem, Israel; 8 Department of Neurology, University Hospital, Hamburg, Germany; 9 Department of Radiology, University Hospital, Hamburg, Germany; University of Manchester, United Kingdom

## Abstract

**Purpose:**

Patients with neurofibromatosis 1 (NF1), NF2, and schwannomatosis are at risk for multiple nerve sheath tumors and premature mortality. Traditional magnetic resonance imaging (MRI) has limited ability to assess disease burden accurately. The aim of this study was to establish an international cohort of patients with quantified whole-body internal tumor burden and to correlate tumor burden with clinical features of disease.

**Methods:**

We determined the number, volume, and distribution of internal nerve sheath tumors in patients using whole-body MRI (WBMRI) and three-dimensional computerized volumetry. We quantified the distribution of tumor volume across body regions and used unsupervised cluster analysis to group patients based on tumor distribution. We correlated the presence and volume of internal tumors with disease-related and demographic factors.

**Results:**

WBMRI identified 1286 tumors in 145/247 patients (59%). Schwannomatosis patients had the highest prevalence of tumors (P = 0.03), but NF1 patients had the highest median tumor volume (P = 0.02). Tumor volume was unevenly distributed across body regions with overrepresentation of the head/neck and pelvis. Risk factors for internal nerve sheath tumors included decreasing numbers of café-au-lait macules in NF1 patients (P = 0.003) and history of skeletal abnormalities in NF2 patients (P = 0.09). Risk factors for higher tumor volume included female gender (P = 0.05) and increasing subcutaneous neurofibromas (P = 0.03) in NF1 patients, absence of cutaneous schwannomas in NF2 patients (P = 0.06), and increasing age in schwannomatosis patients (p = 0.10).

**Conclusion:**

WBMRI provides a comprehensive phenotype of neurofibromatosis patients, identifies distinct anatomic subgroups, and provides the basis for investigating molecular biomarkers that correlate with unique disease manifestations.

## Introduction

The neurofibromatoses, including NF1, NF2, and schwannomatosis, are hereditary tumor predisposition syndromes caused by germline mutations in the *NF1*, *NF2*, and *SMARCB1* tumor-suppressor genes, respectively.[1]–[4] Biallelic inactivation of these tumor-suppressor genes in susceptible cells leads to dysregulation of key cellular machinery, including activation of the Ras pathway (NF1), loss of contact-dependent inhibition of the EGFR pathway (NF2), and perturbation of the SWI-SNF chromatin remodeling complex (schwannomatosis).

These related syndromes have overlapping clinical features and for years, clinicians struggled to differentiate the types of neurofibromatosis. Clinical criteria were established for NF1 and NF2 in 1987 and for schwannomatosis in 2005.[5], [6] These patients share a predisposition to developing benign nerve sheath tumors, including neurofibromas and schwannomas, that are derived from neoplastic Schwann cells. Despite the benign histology of neurofibromas and schwannomas, neurofibromatosis patients have increased mortality due to malignant peripheral nerve sheath tumor (MPNST), glioma, cardiovascular disease, and organ compression by neurofibromas.[7], [8] For NF1 patients, the median age at death is 59 years, compared with 74 years for the general population.[9] For NF2 patients, actuarial survival after diagnosis is 85% at 5 years, 67% at 10 years, and 38% at 20 years.[10] Mortality figures for schwannomatosis have not been reported.

The spectrum of tumor involvement of these disorders is highly variable. In practice, clinicians carefully select the body region to image based on the presence of symptoms and knowledge of disease phenotype (e.g., cranial MRI for vestibular schwannomas in NF2 patients). Imaging of the entire body using traditional regional scans is not possible due to the cost (in time and money) of MRI. For this reason, data regarding the prevalence of whole-body disease patterns from large, multicenter cohorts are lacking.

Whole-body MRI (WBMRI) can evaluate the entire body in a relatively short time without the use of ionizing radiation. We performed an international multicenter study of WBMRI to assess tumor burden of internal nerve sheath tumors in NF1, NF2, and schwannomatosis. Our goal was to identify phenotypic similarities among these related neurogenetic disorders, to identify patterns of tumor involvement, and to relate tumor burden to demographic factors and cutaneous disease manifestations.

## Methods

### Whole-body MRI

We performed WBMRI in patients with NF1, NF2, or schwannomatosis.[5], [11], [12] Inclusion criteria for the study included age ≥18 years of age; diagnosis of NF1, NF2, or schwannomatosis by clinical criteria;[5], [11], [12] and ability to provide written informed consent. Exclusion criteria included inability to undergo MRI because of a medical or psychological condition; presence of a metallic implant; need for general anesthesia; pregnancy; or breast-feeding. Patients were drawn from a convenience sample of patients seen at the Neurofibromatosis clinics at Massachusetts General Hospital and University of Hamburg, Eppendorf, Germany.

### Determination of tumor burden

**Figure 1:**
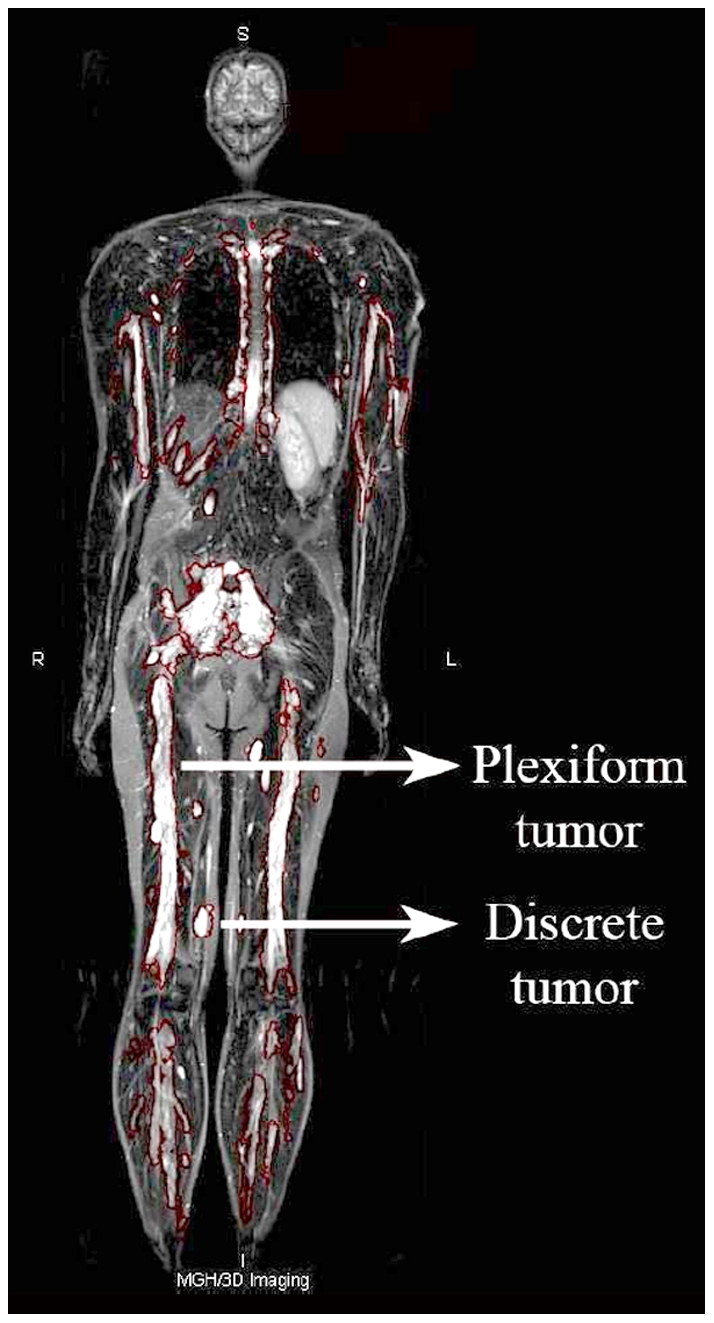
Appearance of internal nerve sheath tumors on Whole-Body MRI. Tumor type was defined according to the radiologic appearance without need for pathological diagnosis. Tumors that were locally circumscribed on MRI scan were classified as circumscribed tumors and those that were invasive or involved multiple nerves were classified as plexiform tumors.

WBMRI was performed once per individual as previously described.[13] MRI scans were first reviewed by a board-certified radiologist who identified the location and appearance (circumscribed vs. plexiform) of each tumor based on its MRI appearance. Tumors that were locally circumscribed on MRI were classified as circumscribed and those that were invasive or involved multiple nerves were classified as plexiform (Figure 1). Pathological diagnosis was not required. Second, each tumor was segmented using computerized volumetry method developed for WBMRI.[13] Third, the study radiologist reviewed the computerized tumor contours. Finally, whole-body tumor burden was determined by recording the number, location, appearance (circumscribed vs. plexiform), and volume of individual tumors for each patient.

### Statistical analysis

#### Whole-body tumor analysis

We calculated descriptive statistics for clinical and demographic factors for each disease group and compared these factors using Kruskal-Wallis test for continuous variables and Chi-square test for categorical variables. We compared whole-body tumor burden among disease groups using Kruskal-Wallis test.

We used whole-body imaging data to identify risk factors associated with internal nerve sheath tumors. We first used logistic regression to analyze the relationship between the presence/absence of internal tumors and clinical/demographic factors. We then used multivariable linear regression to analyze the relationship between tumor volume and clinical/demographic factors in patients with at least one internal tumor. In our analysis, we included clinical and demographic factors previously associated with paraspinal neurofibromas in NF1 and with disease severity in NF2[14]–[16]; we included additional clinical factors to explore their relationship with tumor burden. For multivariable analyses, we included age, gender, inheritance pattern (sporadic vs. familial), number of café-au-lait macules (0 vs. 1–5 vs. 6–15, vs. >15), and history of skeletal abnormalities as covariates in all patients. In NF1 patients only, we included the presence of gliomas, number of cutaneous neurofibromas (0, 1–9, 10–100, 101–500, >500)[17] and number of subcutaneous neurofibromas (0, 1–9, 10–100, 101–500, >500) as covariates. In NF2 patients only, we included the presence of meningiomas and cutaneous schwannomas as covariates. Tumor volumes were natural log-transformed prior to multivariable regression analyses.

#### Distribution analysis

We calculated the absolute and relative tumor volume in six anatomic regions (head/neck, chest, abdomen, pelvis, arms, and legs) in patients with internal tumors. We then quantified the distribution of tumor volume across body regions for each patient (i.e., regionality of tumor burden) using the Gini coefficient.[18] A low Gini coefficient indicates a more even distribution across body parts, with a value of 0 corresponding to uniform distribution of tumor volume across body regions; higher Gini coefficients indicate a more unequal distribution with a value of 1 corresponding to concentration of tumor volume to a single body region.

We then identified specific body regions preferentially affected by tumors. We adjusted our analysis to account for known differences in the size of each body part.[19] In this analysis, the null hypothesis was that the percentage of whole-body tumor volume in each body part would be equivalent to the percentage of whole-body volume of each body part. To determine the average volume of each body part, we measured the volume of body parts in 5 male and 5 female patients selected at random. We then used a bootstrap method to test for significance of the Manhattan distance between the predicted distribution and the observed average distribution.

We next used unsupervised cluster analysis to sort patients into groups based on anatomic predisposition. Tumor volumes across all regions were combined and scaled for each patient; scores were then analyzed by agglomerative hierarchical clustering. We partitioned the resulting dendrogram and assessed possible associations between the resulting groups and the following variables by a series of Chi-square tests: diagnosis, number of café-au-lait macules, presence of skeletal abnormalities, presence of gliomas, inheritance pattern, presence of cutaneous tumors, and gender. We assessed the variable age using Kruskal-Wallis test.

All statistical calculations were performed with SAS software (version 9.2, SAS Institute Inc, NC, USA) and the statistical programming language R.[20] The distribution and cluster analyses were designed post-hoc. The study was approved by the institutional review boards at Massachusetts General Hospital (Partners Human Research Committee); University of Hamburg, Eppendorf, Germany (Ethics Committee of the Chamber of Physicians, Hamburg); and the Department of Defense (Human Research Protection Office). Written informed consent was obtained from all participants.

## Results

### Study patients

Between January 2007 and November 2010, a total of 247 patients underwent WBMRI. The cohort included 141 NF1 patients (57%), 55 NF2 patients (22%), and 51 schwannomatosis patients (21%). Baseline demographic and clinical characteristics of the cohort are shown in Tables 1 and 2, respectively.

**Table 1:** Demographic features of the 247 patients who underwent whole-body MRI.

Demographic features	Neurofibromatosis 1N = 141	Neurofibromatosis 2N = 55	SchwannomatosisN = 51	P-value
Mean age (years)	38.5	39.1	48.5	< 0.001
Mean age at diagnosis (years)	14.5	29.1	41.8	< 0.001
Mean age at diagnosis of first internal tumor (years)	18.7	28.1	37.4	< 0.001
Sex (%)-male	46.8%	41.8%	51.0%	.64
Mean height (cm)	167	167	170	.006
Mean weight (kg)	71	69	81	< 0.001
Highest degree				
High school or less	45%	27%	27%	
College or higher	55%	73%	73%	
Inheritence				.17
Familial	42 (29.8%)	14 (25.5%)	8 (15.7%)	
Sporadic	98 (69.5%)	41 (74.5%)	41 (80.4%)	
Unknown	1 (0.7%)	0 (0%)	2 (3.9%)	

**Table 2:** Clinical features of the 247 patients who underwent whole-body MRI.

Neurofibromatosis 1 (n = 141)		Neurofibromatosis 2 (n = 55)		Schwannomatosis (n = 51)	
**Medical history**					
Plexiform neurofibroma	76/140 (54%)	Vestibular schwannoma	54/55 (98%)	Vestibular schwannoma	0/51 (0%)
Spinal neurofibroma	33/139 (24%)	Spinal schwannoma	30/52 (58%)	Spinal schwannoma	27/50 (54%)
Optic glioma	16/141 (11%)	Internal schwannoma	19/52 (37%)	Internal schwannoma	30/51 (59%)
Non-optic glioma	12/141 (8.5%)	Meningioma	29/54 (54%)	Meningioma	0/48 (0%)
GIST	3/137 (2%)	Ependymoma	19/53 (36%)	Ependymoma	0/44 (0%)
Pheochromocytoma	5/137 (4%)	Hearing loss	52/54 (96%)	Hearing loss	7/51 (14%)
≥ 2 Lisch nodules	62/113 (55%)	Tinnitus	39/55 (71%)	Tinnitus	3/51 (6%)
ADHD	20/139 (14%)	Epiretinal membrane or retinal hamartoma	4/46 (9%)	Epiretinal membrane or retinal hamartoma	0/30 (0%)
Learning disability	43/138 (31%)	Cataracts	13/46 (28%)	Cataracts	5/30 (17%)
Seizures	7/141 (5%)	Seizures	8/55 (15%)	Seizures	1/51 (2%)
Skeletal complication	44/140 (31%)	Skeletal complication	6/55 (11%)	Skeletal complication	3/51 (6%)
Scoliosis	40 (28%)	Scoliosis	6 (11%)	Scoliosis	3 (6%)
Pseudarthrosis	1 (1%)	Pseudoarthrosis	0 (0%)	Pseudoarthrosis	0 (0%)
Sphenoid wing dysplasia	1 (1%)	Sphenoid wing dysplasia	0 (0%)	Sphenoid wing dysplasia	0 (0%)
Bone cysts	2 (1%)	Bone cysts	0 (0%)	Bone cysts	0 (0%)
**Physical examination**					
≥ 6 cafe-au-lait macules	111/140 (79%)	≥ 6 cafe-au-lait macules	3/55 (5%)	≥ 6 cafe-au-lait macules	0/51 (0%)
Skin fold freckling	125/141 (89%)				
Cutaneous neurofibromas	119/141 (84%)	Cutaneous schwannomas	21/55 (38%)	Cutaneous schwannomas	9/51 (18%)
< 10	21 (15%)	1–5	16 (29%)	1–5	9 (18%)
10–100	46 (33%)	6–10	4 (7%)	6–10	
101–500	33 (23%)	>10	1 (2%)	>10	
> 500	19 (13%)				
Subcutaneous neurofibromas	110/141 (78%)	Subcutaneous schwannoma	15/55 (27%)	Subcutaneous schwannomas	18/51 (35%)
< 10	51 (36%)	1–5	12 (22%)	1–5	13 (25%)
10–100	38 (27%)	6–10	3 (5%)	6–10	3 (6%)
101–500	17 (12%)	>10	0 (0%)	>10	2 (4%)
> 500	4 (3%)				

### Whole-body tumor count and volume

We identified a total of 1286 nerve sheath tumors (528 plexiform and 758 circumscribed tumors) comprising 65,423 ml in 145/247 patients (59%). Internal tumors were more common in schwannomatosis patients (71%) than in NF2 patients (45%) (p = 0.01); the prevalence in NF1 patients (60%) was not significantly different than either schwannomatosis or NF2 patients (p>0.05). Plexiform tumors were found in all three groups, more commonly in NF1 patients (40%) than in NF2 and schwannomatosis patients (18% and 14%, respectively) (p = 0.01). Among 76 patients who denied a history of internal nerve sheath tumors at enrollment, 30 (39%) were found to have tumors on WBMRI. This group included 22/52 NF1 patients (42%), 1/16 NF2 patients (6%), and 7/8 schwannomatosis patients (88%).

**Figure 2:**
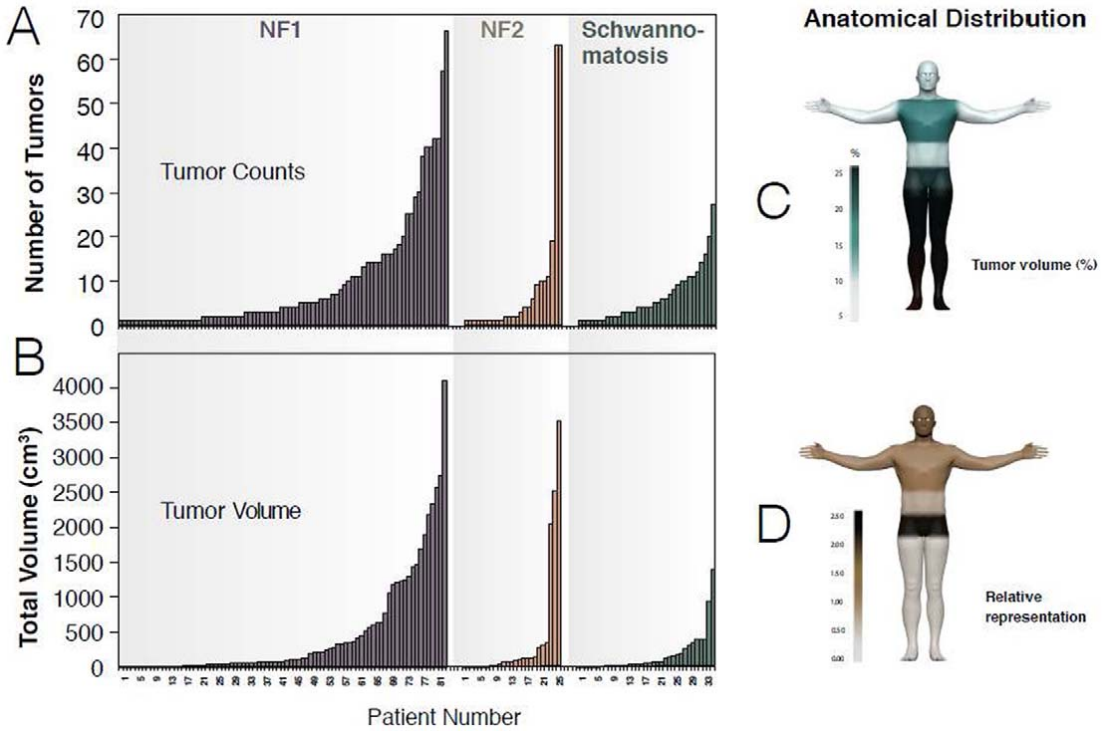
Number, volume, and anatomic distribution of internal nerve sheath tumors. Waterfall plot of tumor count (panel A) and tumor volume (Panel B) in 145 patients with at least one internal tumor. Anatomic distribution of relative tumor volume given as a percentage of whole-body volume (Panel C) and corrected for volume per body part (Panel D).

**Figure 3:**
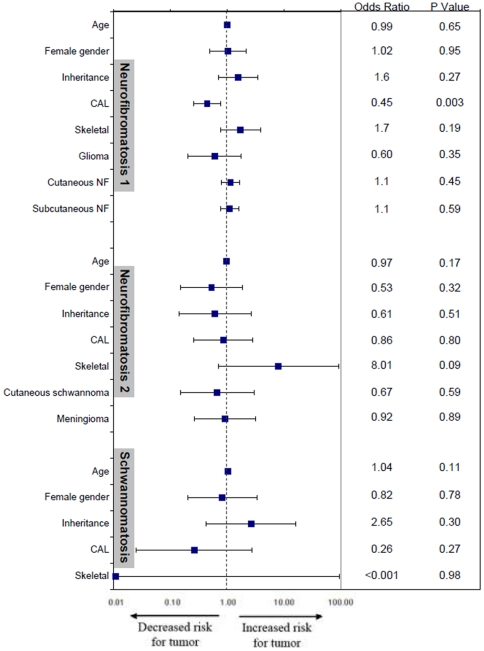
Odds ratios for the presence of internal nerve sheath tumors. Odds ratio for the presence of internal nerve sheath tumors in NF1, NF2, and schwannomatosis patients, according to clinical and demographic characteristics. Odds ratios and 95% confidence intervals were calculated with the use of logistic regression analsysis. Squares indicate odds ratios and horizontal lines indicate 95% confidence intervals. P values are for the odds ratios.

In patients with internal tumors on WBMRI, tumor count ranged from 1 to 69; the distribution of tumor count per patient showed an exponential decline (Figure 2A). In patients with at least one tumor, the median number of tumors did not differ by diagnosis (Table 3). In logistic regression analysis, the presence of internal nerve sheath tumors was correlated with decreasing number of café-au-lait macules in NF1 patients (P = 0.003) and with history of skeletal abnormalities in NF2 patients (P = 0.09) (Figure 3). All other clinical factors had P-values >0.10, the level of significance used for this exploratory correlative analysis.

**Table 3:** Whole-body tumor number and volume in patients with at least 1 internal tumor.

	Characteristic	NF1N = 84	NF2N = 25	SchwannomatosisN = 36	p-value
**All tumors**	Median number of tumor per patient	4.5	2.0	4.0	0.21
	Median tumor volume per patient – ml	107.9	69.5	39.4	0.02
**Circumscribed tumors**	Median number of tumor per patient	3.0	2.0	5.0	0.30
	Median tumor volume per patient – ml	29.9	38.8	31.3	0.53
**Plexiform tumors**	Median number of tumor per patient	3.0	3.0	3.0	0.70
	Median tumor volume per patient – ml	205.2	124.6	107.5	0.76

In patients with internal tumors, whole-body tumor volume per patient ranged from 1.2 ml to 9106.1 ml; the distribution of total tumor volume showed a progressive decline in frequency as tumor volume increased (Figure 2B). The median whole-body tumor volume for all groups was 83.0 ml and differed among diagnosis groups (p = 0.02, Table 3). Although 41% (528/1286) of lesions were plexiform in appearance, these tumors contributed 78% of the total tumor volume: the median tumor volume was 29.4 ml per plexiform tumor and 6.7 ml per circumscribed tumor. In multivariable analysis, increased tumor volume was correlated with female gender (P = 0.05) and with presence of subcutaneous neurofibromas (P = 0.03) in NF1 patients, with the absence of cutaneous schwannomas in NF2 patients (P = 0.06), and with increasing age in schwannomatosis patients (P = 0.10). All other clinical factors had P-values >0.10.

### Distribution analysis of internal nerve sheath tumors

The median Gini coefficient was 0.84 (range, 0.26 to 1.0), suggesting that tumor volume within patients was not evenly distributed across body parts. The majority of patients (122/144, 85%) had high Gini coefficients (≥0.67) indicating that tumor volume was concentrated in a limited region. Seventeen patients (12%) had moderate Gini coefficients (0.34–0.66), and just 5 patients (3%) had low Gini coefficients (≤0.33) indicating even distribution of tumor volume across body parts. The complete collection of WBMRI with tumor volumes and Gini coefficients can be viewed at www.wholebodymri.org.

Overall, the legs harbored the greatest volume of tumors (31%), followed by the pelvis (22%), thorax (17%), abdomen (13%), arms (11%) and head/neck (6%) (Figure 2C). When compared with the relative volume of each body part, the distribution showed that the head/neck and pelvis were over-represented while the legs were under-represented (p<0.001, Figure 2D).

**Figure 4:**
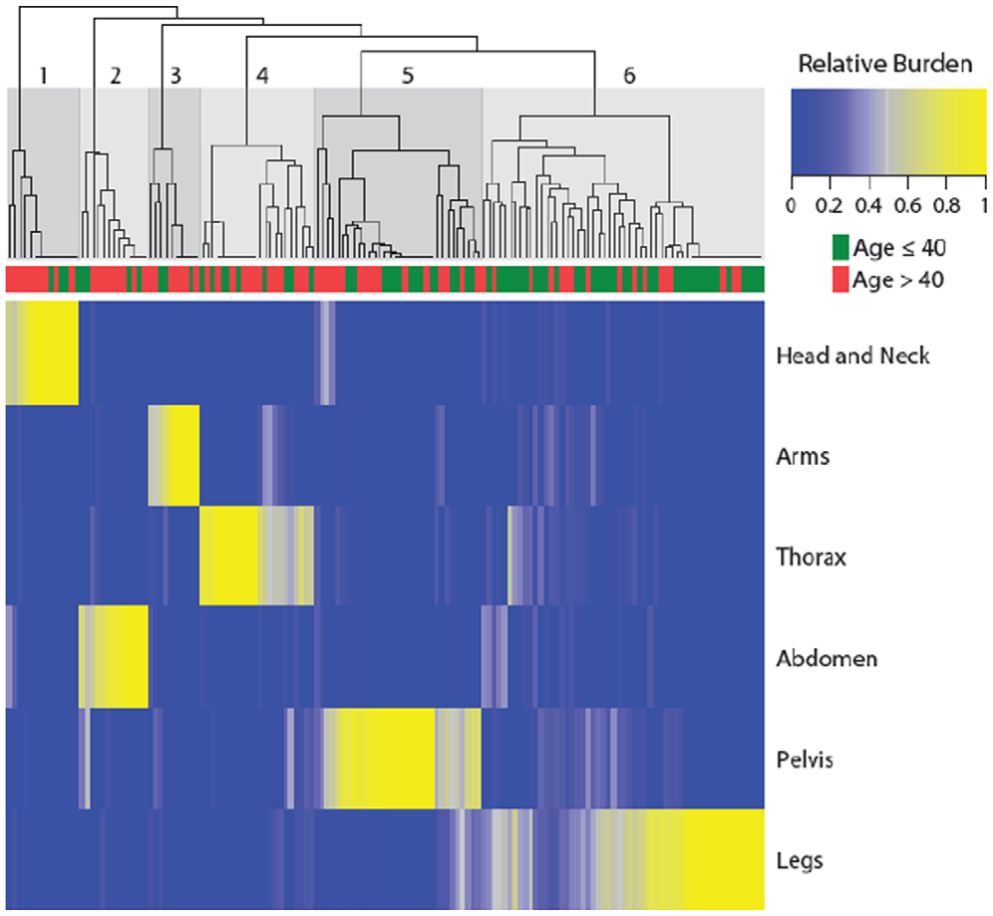
Results of unsupervised hierarchical clustering of patients based on relative tumor volume across body regions. (This figure is an interactive display. It is currently available for at www.wholebodymri.org). Unsupervised hierarchical clustering of relative tumor volume across body region (head/neck, trunk, extremities) for 145 subjects with internal nerve sheath tumors. Total tumor volumes were combined and scaled for each patient. Regions with higher tumor burden are shown in yellow. Individual patients are represented as columns and the maximum intensity projection (MIP) of the whole-body MRI scan is shown to the right of the clustering figure. For each patient, the Gini coefficient is shown in the lower left and the whole-body tumor volume in the lower middle.

Clustering of our data set revealed distinct groups of patients based on predominant tumor location. The most common pattern was leg-predominant (54/145, 37%), followed by pelvis-predominant (32/145, 22%), thorax-predominant (22/145, 15%), head/neck-predominant (14/145, 10%), abdomen-predominant (13/145, 9%), and arms-predominant (10/145, 7%) (Figure 4).

**Figure 5:**
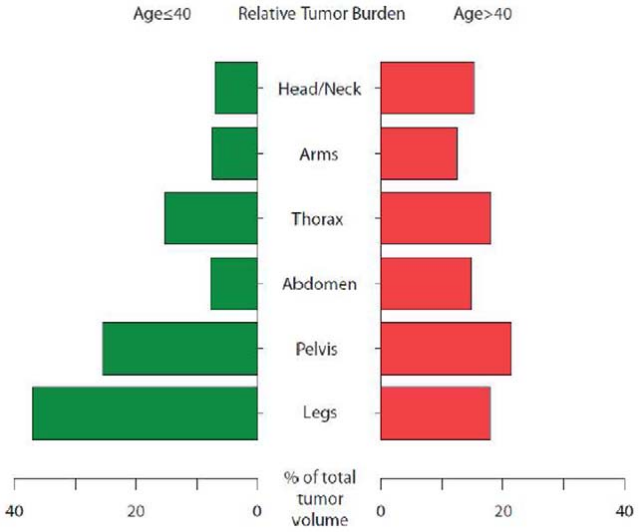
Relative tumor burden of patients younger and older than 40. Patients older than 40 are represented in red; patients 40 or younger are shown in green. These two patient subpopulations differed significantly in relative tumor burden along the craniocaudal axis (p = 0·003 by permutation test) with patients 40 or younger having a higher percentage of tumor volume in the pelvis and legs than patients over 40.

Clustering was associated with age (p = 0.06) and with inheritance pattern (sporadic vs. familial) (P = 0.1). Patients were then dichotomized according to median age at diagnosis (≤20 years vs. >20 years), median age at WBMRI (≤40 years vs. >40 years), and inheritance pattern for comparison of relative tumor volume across body regions. There was no significant difference between sporadic and familial patients or between patients diagnosed before or after 20. However, patients ≤40 years had a higher proportion of tumor volume in the pelvis and legs compared those >40 (Figure 5). Clinical variables which did not differ significantly among clusters included diagnosis, numbers of café-au-lait macules, skeletal abnormalities, gender, number of cutaneous neurofibromas, and presence of gliomas (p>0.1).

## Discussion

In this study, we used WBMRI to examine internal tumor burden in three closely related tumor-suppressor syndromes. Our findings corroborate that WBMRI can detect internal tumors in a far higher percentage of patients than conventional, regional imaging methods.[21] For example, the prevalence of plexiform or spinal nerve sheath tumors on regional MRI or CT scan ranges from 16%–39% in NF1 patients.[17], [22]–[25] Using WBMRI, we found that 60% of such patients have internal nerve sheath tumors. As expected, patients without a known history of internal tumor had high rates of lesions on WBMRI, indicating that asymptomatic tumors are common in this population [25].

Because WBMRI can detect even relatively small or asymptomatic tumors in all body regions, it provides a more comprehensive picture of tumor burden in patients and, for the first time, allows for analysis of tumor distribution across body parts. We used novel approaches to describe this distribution, including Gini coefficient to measure the regionality of tumor burden and clustering analysis to sort patients into groups based on tumor predilection. Our results show that internal nerve sheath tumors, like dermal neurofibromas, are not randomly or evenly distributed across body parts (see www.wholebodymri.org). Instead, particular body regions appear to be preferentially affected, while other body regions are relatively spared.

There are multiple processes that might explain why the pelvis and head/neck are particularly affected by internal tumors or why younger patients have a greater proportion of internal tumor volume in the pelvis and legs. Genetic mosaicism is well documented in neurofibromatosis and may be diagnosed when patients display disease features that are restricted to portions of the body (e.g., segmental findings).[26], [27] Alternatively, there may be tissue-specific biological factors in the affected regions that are permissive for tumor formation. It is increasingly clear that neurofibroma formation requires a microenvironment containing bone marrow-derived cells that are heterozygous at *Nf1*.[28] These biological factors could be important during development (e.g., for congenital lesions like plexiform neurofibromas) or post-natally (e.g., for non-congenital lesions like vestibular schwannomas). Genetically engineered mouse (GEM) models of NF1 and NF2 develop nerve sheath tumors in restricted locations that differ from humans.[29]–[31] Subsequent investigations of molecular biomarkers in both GEM and human tissue may help identify biological factors involved in these unique disease manifestations.

Overall, tumor burden and patterns of regionality were similar for the three tumor-suppressor syndromes. We observed a high prevalence of nerve sheath tumors in schwannomatosis patients (71%), NF1 patients (60%), and NF2 patients (42%). There was no difference in the median number of tumors but there was greater median tumor volume in NF1 patients than in NF2 or schwannomatosis patients. This difference was due to the increased prevalence of larger plexiform tumors in this population. The overall similarity in tumor burden among these conditions is striking given the diverse functions of the *NF1*, *NF2*, and *SMARCB1* tumor-suppressor genes. Inactivation of *NF1* leads to upregulation of RAS signaling [32]; inactivation of *NF2* leads to dysregulation of cell surface receptors and intercellular signaling, and to disinhibition of the E3 ubiquitin ligase CRL4(DCAF1)[33], [34]; and inactivation of *SMARCB1* leads to dysregulation of the SWI-SNF chromatin remodeling complex [35]. Current laboratory models do not explain the precise interaction between these pathways, but can be used to explore whether these separate pathways converge upon a final common pathway in Schwann cells.

Having an accurate phenotype of patients not only provides a basis for future research investigations, but has important implications for clinical management of NF patients. Our data identifies clinical risk factors for internal nerve sheath tumors in the neurofibromatoses; this information builds upon established genetic factors that influence tumor burden, such as *NF1* gene microdeletions (increased tumor burden) [36] and mosaicism for the NF2 gene (decreased tumor burden).[27] In NF1 patients, decreasing number of cafe-au-lait macules correlated with the presence, but not volume, of internal neurofibromas (Figure 3). This finding, which was reported recently,[14] was not explained by increasing age since this variable was included in the multivariate model. An inverse relationship between the presence of neurofibromas and café-au-lait macules – the two cardinal features of NF1 – has been reported in rare variants of NF1. Patients with spinal neurofibromatosis have multiple spinal neurofibromas without café-au-lait macules whereas patients with a 3-bp inframe deletion in exon 17 of the *NF1* gene have café-au-lait macules without neurofibromas.[37] Our data suggests that this inverse relationship may apply to a broader subset of patients than previously recognized. Further research should examine differential effects of germline *NF1* mutations on melanocytes and Schwann cells since both are derived from neural crest cells.

Increasing numbers of subcutaneous tumors correlated with increasing neurofibroma volume in NF1 patients, corroborating previous reports [38] and presumably reflecting their shared biologic underpinnings with internal tumors. We also found that women with NF1 have greater tumor burden, on average, than men. Although the biological basis for this finding is not known, some studies have suggested that sex hormones may stimulate tumor growth. For example, tumor number and size increase in women during pregnancy,[17], [39] and in women using contraception with high doses of synthetic progesterone.[40] In addition, laboratory studies have implicated progesterone in neurofibroma progression.[41] Additional studies on the effect of sex hormones on tumor formation and growth are warranted to clarify this finding.

Our data identify scoliosis and decreasing numbers of cutaneous schwannomas as risk factors for internal schwannomas in NF2. While the presence of internal schwannomas correlated with scoliosis, it did not correlate with established markers of disease severity such as spinal tumors or meningiomas.[15], [16] For this reason, we suspect that scoliosis may result from spinal tumors rather than being a predisposing factor. Unexpectedly, whole-body tumor volume in NF2 patients was inversely correlated with the number of cutaneous schwannomas.[15] If this finding is confirmed, it further highlights the importance of different microenviroments in tumor formation. In schwannomatosis patients, no risk factors were identified for the presence of internal schwannomas although increasing age correlated with whole-body tumor volume. This finding may explain, in part, the late age at onset of symptoms compared to NF1 and NF2 [5].

The limitations of our study include the use of a sample of convenience at two large referral centers and the lack of children in the study population. For this reason, our study population does not represent all patients with neurofibromatosis or schwannomatosis. Additionally, coverage of the legs and arms by WBMRI may be incomplete in individuals who are tall or heavy. Our study did not include serial WBMRI scans of individual patients and therefore cannot determine the rate by which tumor count or volume changed with time. Future longitudinal studies of WBMRI in NF patients should quantify changes in tumor burden over time to better understand tumor progression.

In conclusion, in this large prospective international study, we found high rates of internal nerve sheath tumors in neurofibromatosis patients and showed that tumors were non-randomly distributed across body parts. These results provide valuable information about risk factors for internal nerve sheath tumors and raise new biological questions for future research. The addition of WBMRI to careful phenotyping represents a powerful approach to studying hereditary tumor predisposition syndromes and other complex genetic syndromes in humans.
